# Dietary phenotype and advanced glycation end-products predict WTC-obstructive airways disease: a longitudinal observational study

**DOI:** 10.1186/s12931-020-01596-6

**Published:** 2021-01-18

**Authors:** Rachel Lam, Sophia Kwon, Jessica Riggs, Maria Sunseri, George Crowley, Theresa Schwartz, Rachel Zeig-Owens, Hilary Colbeth, Allison Halpren, Mengling Liu, David J. Prezant, Anna Nolan

**Affiliations:** 1 Department of Medicine, Division of Pulmonary, Critical Care and Sleep Medicine, New York University, School of Medicine, New York, NY USA; 2 Division of Biostatistics, Departments of Population Health, New York University School of Medicine, New York, NY USA; 3 Fire Department of New York, Bureau of Health Services, Brooklyn, NY USA; 4 Pulmonary Medicine Division, Department of Medicine, Montefiore Medical Center and Albert Einstein College of Medicine, Bronx, NY USA; 5 Department of Environmental Medicine, New York University, School of Medicine, New York, NY USA; 6 Department of Medicine, Division of Pulmonary, Critical Care and Sleep, New York University, School of Medicine, New Bellevue, 16 S Room 16 (Office), 16N Room 20 (Lab), 462 1st Avenue, New York, NY 10016 USA

**Keywords:** Metabolic syndrome, Nutrition, Diet

## Abstract

**Background:**

Diet is a modifier of metabolic syndrome which in turn is associated with World Trade Center obstructive airways disease (WTC-OAD). We have designed this study to (1) assess the dietary phenotype (food types, physical activity, and dietary habits) of the Fire Department of New York (FDNY) WTC-Health Program (WTC-HP) cohort and (2) quantify the association of dietary quality and its advanced glycation end product (AGE) content with the development of WTC-OAD.

**Methods:**

WTC-OAD, defined as developing WTC-Lung Injury (WTC-LI; FEV_1_ < LLN) and/or airway hyperreactivity (AHR; positive methacholine and/or positive bronchodilator response). Rapid Eating and Activity Assessment for Participants-Short Version (REAP-S) deployed on 3/1/2018 in the WTC-HP annual monitoring assessment. Clinical and REAP-S data of consented subjects was extracted (7/17/2019). Diet quality [low-(15–19), moderate-(20–29), and high-(30–39)] and AGE content per REAP-S questionnaire were assessed for association with WTC-OAD. Regression models adjusted for smoking, hyperglycemia, hypertension, age on 9/11, WTC-exposure, BMI, and job description.

**Results:**

N = 9508 completed the annual questionnaire, while N = 4015 completed REAP-S and had spirometry. WTC-OAD developed in N = 921, while N = 3094 never developed WTC-OAD. Low- and moderate-dietary quality, eating more (processed meats, fried foods, sugary drinks), fewer (vegetables, whole-grains),and having a diet abundant in AGEs were significantly associated with WTC-OAD. Smoking was not a significant risk factor of WTC-OAD.

**Conclusions:**

REAP-S was successfully implemented in the FDNY WTC-HP monitoring questionnaire and produced valuable dietary phenotyping. Our observational study has identified low dietary quality and AGE abundant dietary habits as risk factors for pulmonary disease in the context of WTC-exposure. Dietary phenotyping, not only focuses our metabolomic/biomarker profiling but also further informs future dietary interventions that may positively impact particulate matter associated lung disease.

## Background

Diet and obesity play a role in the development of obstructive airways disease (OAD) [1–3]. Diets focused on reducing inflammation and increasing vegetable and fish consumption reduced the risk of chronic obstructive pulmonary disease (COPD), whereas diets with increased pro-inflammatory advanced glycation end products (AGE) were associated with disease [4–7]. Low-calorie dietary interventions yielded weight loss and improved lung function in obese asthmatics [8]. The health benefits of weight loss, increased high density lipoprotein (HDL), and decreased triglyceride, have been extensively studied [9–11]. Specifically, Mediterranean diets characterized by high consumption of fruits, vegetables, and fish, were associated with lower COPD, whereas, western diets were significantly associated with higher risk of newly diagnosed COPD [12–16].

Metabolic syndrome (MetSyn) is a risk factor of cardiovascular, lung disease, and World Trade Center-OAD (WTC-OAD) [15, 16]. MetSyn affects over 30% of US adults and 23% of participants in the Fire Department of New York (FDNY) WTC-Health Program (WTC-HP) program [12–19]. Furthermore, metabolic biomarkers, elevated BMI, and a > 2 kg/m^2^ BMI increase predicted WTC-OAD [16, 20–23].

Since high-caloric diets are key contributors to MetSyn, nutritional interventions to potentially reverse pulmonary dysfunction have been studied [14–16]. Our in vitro and in vivo models identified that the receptor for AGE (RAGE) is associated with lung dysfunction after WTC-particulate matter (WTC-PM) exposure. Specifically, RAGE deficient WTC-PM exposed mice were protected against WTC-OAD [24–26]. Dietary and endogenous AGEs can impact signaling pathways such as those in inflammatory diseases [27]. Despite evidence that diet and obesity are risks, studies have suggested obesity may have a protective effect on survival and lung function in COPD [28, 29]. Therefore, to further clarify the effect of diet on lung disease in our WTC-exposed cohort, we studied their dietary patterns.

The Rapid Eating and Activity Assessment for Patients (REAP) and its short version (REAP-S) are advantageous over other independently developed and validated food questionnaires in the primary care setting because of their brevity and ability to quickly evaluate targeted food categories, potential barriers to high dietary quality, and dietary habits [30–43]. REAP-S score has also correlated with other questionnaires investigating OAD [44–48].

To inform our understanding of how diet is a modifier of WTC-OAD, we utilized REAP-S to assess dietary quality and estimate intake of foods such as fat, cholesterol, sugar, and meats and correlated it to disease outcome [30, 43, 47–49]. This study also prospectively evaluated potential barriers to high dietary quality, dietary habits, and food group stratification for AGE content. We hypothesized that WTC-exposed first responders with poor dietary quality and increased AGE content were more likely to have WTC-OAD at any timepoint after 9/11/2001 (9/11).

## Methods

### Study design

**Fig. 1:**
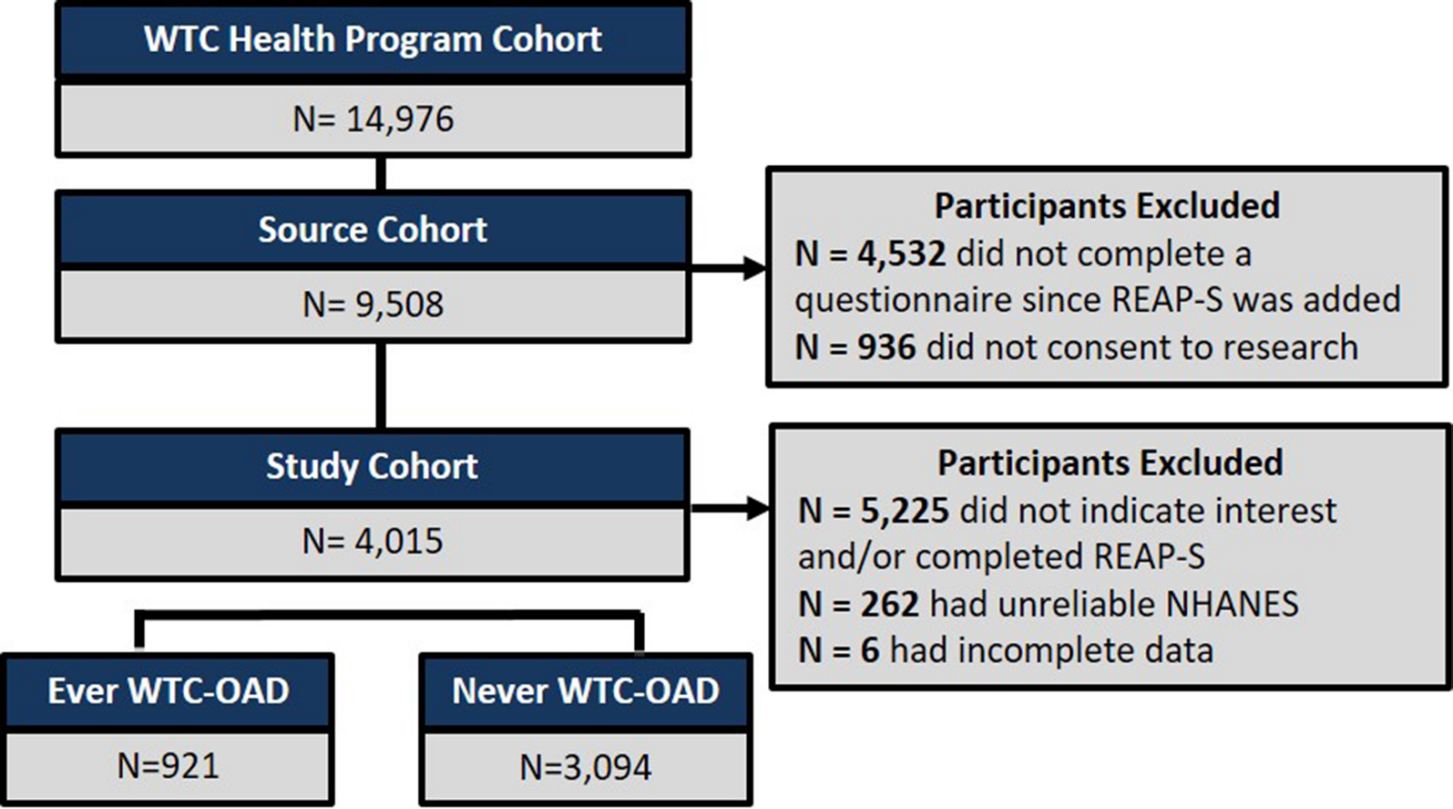
Study Design/Consort Diagram. FDNY Rescue/recovery Workers Exposed to World Trade Center Particulates

This observational study targeted N = 14,976 WTC-HP enrollees that had annual monitoring exams, including physical health and mental health questionnaires, Fig. 1. REAP-S was implemented in the annual questionnaire on March 1, 2018 and continuously accrued until July 17, 2019. Two questions were used to gauge interest in answering the REAP-S and screen for willingness to change diet, Additional file 1: Table S1.

Source cohort (N = 9508) completed an annual health questionnaire and consented to further physical health research. Subjects were further screened for the study cohort (N = 4015) if they met the following criteria: (i) completed REAP-S (ii) had reliable National Health and Nutrition Examination Survey (NHANES) and (iii) had complete clinical data. Demographic characteristics, clinical data, 9/11 exposure characteristics, questionnaire answers, and lung function testing were obtained from the FDNY WTC-HP electronic medical record (EMR). Study approved by the Montefiore Medical Center/Albert Einstein College of Medicine IRB #07-09-320.

### WTC-OAD case definitions

Cases of WTC-OAD had either WTC-Lung Injury (WTC-LI; FEV_1_ < LLN) and/or Airway Hyperresponsiveness (AHR; positive methacholine or positive bronchodilator testing) at any time point post-9/11 (N = 921) [16, 16, 21, 21–24, 50–62]. Cases of WTC-OAD were compared to N = 3094 without WTC-OAD at any time after 9/11.

Our group has utilized FEV_1_ to define WTC-LI [16, 16, 21–23, 53, 59, 60, 63–65]. FEV_1_ was measured prior to 9/11/2001 and is still performed at every FDNY-HP visit. This gives a comprehensive measure of changing lung function over time. Using abnormal FEV_1_ as an outcome improves generalizability of our findings since it is a readily available measure that doesn’t require costly instrumentation. A vast majority of the WTC cohort had airflow obstruction [51]. Deterioration of FEV_1_ < LLN is a robust disease definition, correlates with mortality and somewhat with OAD outcomes (severity leading to hospitalizations, exercise ability and measures of quality of life measures) [66–73]. Using FEV_1_ as a single measure of lung function could lead to non-differential misclassification. Since FEV_1_ is reduced in both restriction and obstruction, FEV_1_ < LLN does not distinguish between the two. In spite of the potential for non-differential information bias, using FEV_1_ < LLN has yielded strong biomarkers-disease associations [74, 75]. Therefore, FEV_1_ < LLN is a surrogate for obstruction in WTC-exposed firefighters and was how we defined WTC-LI [51, 76, 77].

### Nutritional assessment

REAP-S was scored and summed as per guidelines, Table 2 and Additional file 1: Table S1 [43]. REAP-S scores can range from 15–39, and higher quantities represent dietary quality characterized by optimal intake of fruits, vegetables, and whole grains and decreased intake of sugary foods, processed meats, and fried foods. Scores were categorized into low-dietary [15–19], moderate-dietary [16, 20–28], and high-dietary [29–38] quality, Table 3. Additionally, REAP-S questions were assessed as distinct food categories.

*AGE quantification* (kU/serving) in food groups represented in REAP-S was compiled to a representative value per food group, Additional file 2: Figure S2 [78].

### Statistics

Primary data storage/analyses performed with SPSS 25 (IBM) and Prism 8 (Graphpad). Mean ± standard deviation (SD) expressed as continuous variables. Paired sample t-tests compared clinical parameters at two time points—first measurement post-9/11 and at REAP-S administration; student t-tests compared clinical data of those with WTC-OAD to those who never developed WTC-OAD. One-way ANOVA was used in a subgroup analysis of lung function and dietary quality. Counts and percentages describe categorical variables and compared groups using χ^2^-test.

Arrival time and smoking was self-reported and collected through the annual questionnaires/EMR. Arrival time data, used as a proxy for WTC-particulate matter (WTC-PM) exposure, was categorized into a dichotomous variable of “arrived at the site in the morning of 9/11” or “anytime thereafter” [79]. Smoking data was dichotomous representing ever or never smokers [51, 59, 64, 76, 77, 80–85].

*Modeling using* Multivariable logistic regression estimated association of AGE abundancy, REAP-S scores, and the development of WTC-OAD. All models were adjusted for smoking, age at September 11, 2001, exposure intensity, BMI, and job description. We assumed that dietary habits remain relatively constant over time [86–88]. Models of WTC-OAD using components of REAP-S were corrected for multiple comparisons by Bonferroni, p < 0.005. For all else, p was significant if < 0.05 and omnibus testing assessed variance of data.

## Results

### FDNY nutrition cohort characteristics

There were no significant demographic differences between the source cohort (N = 9508) and the study cohort (N = 4015/9508; 42.23%). Out of the total subjects with WTC-OAD (N = 921), 586 subjects (63.62%) had WTC-LI only, 197 subjects (21.39%) had AHR only, and 138 subjects (14.98%) had both WTC-LI and AHR. Within those with AHR (N = 335), 126 (37.61%) had a positive bronchodilator, 175 (52.24%) had a positive methacholine, and 34 (10.15%) had both.

Subjects with WTC-OAD were more likely to be retired, member of the emergency medical services (EMS) rather than firefighter, and exposed the morning of 9/11 when compared to those who never developed WTC-OAD (p < 0.001), Table 1. Of note, age at 9/11, smoking status, and race were no different in the WTC-OAD and never WTC-OAD populations, Table 1.

**Table 1:** Demographic and clinical data

Measures	Study cohort N = 4015	WTC-OAD		p
		Ever N = 921	Never N = 3094	
Demographics				
Age on 9/11	40.55 (7.40)	40.64 (7.12)	40.53 (7.48)	0.68
Retired at exam	3077 (76.60%)	765 (83.10%)	2312 (74.70%)	< 0.001
Firefighter	3637 (90.60%)	806 (87.50%)	2831 (91.50%)	< 0.001
Ever smokers	1291 (32.20%)	315 (34.20%)	976 (31.50%)	0.13
Caucasian	3819 (95.10%)	886 (96.20%)	2933 (94.80%)	0.08
Arrived morning of 9/11	679 (16.90%)	183 (19.90%)	496 (16%)	< 0.001
1st Post-9/11				
FEV_1%Pred_	96.92 (14.02)	83.93 (13.10)	100.79 (11.78)	< 0.001
FVC_%Pred_	92.36 (12.15)	83.58 (11.58)	94.88 (11.09)	< 0.001
BMI kg/m^2^	29.02 (3.78)	29.59 (4.16)	28.84 (3.65)	< 0.001
Systolic BP mmHg	117.91 (14.37)	119.27 (14.58)	117.51 (14.28)	< 0.001
Diastolic BP mmHg	74.15 (9.26)	75.01 (9.40)	73.89 (9.20)	< 0.001
HDL mg/dL	48.02 (11.58)	47.54 (11.80)	48.16 (11.51)	0.20
LDL mg/dL	126.54 (36.08)	127.08 (34.65)	126.39 (36.5)	0.63
Cholesterol (total) mg/dL	208.72 (43.89)	212.07 (59.80)	207.73 (37.84)	< 0.001
Triglyceride mg/dL	179.91 (131.57)	191.93 (137.60)	176.34 (129.52)	< 0.001
At REAP-S				
FEV_1%Pred_	93.01 (14.33)	77.89 (14.18)	97.44 (10.95)	< 0.001
FVC_%Pred_	91.59 (12.51)	80.42 (12.92)	94.86 (10.31)*	< 0.001
BMI kg/m^2^	30.34 (4.87)	31.15 (5.49)	30.09 (4.65)	< 0.001
Systolic BP mmHg	126.06 (12.99)	127.19 (13.10)	125.72 (12.94)	< 0.001
Diastolic BP mmHg	78.70 (8.38)	79.28 (8.10)	78.53 (8.46)	0.020
HDL mg/dL	53.76 (14.61)	53.01 (14.68)	53.97 (14.58)	0.18
LDL mg/dL	116.93 (33.64)	113.76 (34.16)	117.82 (33.45)	0.001
Cholesterol (total) mg/dL	194.91 (39.45)	191.33 (41.32)	195.91 (38.86)	0.020
Triglyceride mg/dL	129.16 (223.39)	135.54 (80.60)	127.38 (249.01)	0.46

All available measures are Mean (SD) or N (%)

p-values displayed represent comparisons between ever/never WTC-OAD by Student’s t-tests

^*^ FVC_%Pred_ for Never WTC-OAD comparing 1st Post-9/11 and REAP-S by Paired t-tests was not significant, all other measures were p < 0.05

### Clinical measures

Time to reach WTC-OAD case definition was (mean ± SD) 6.37 ± 7.23 years for the study cohort. For both ever WTC-OAD cases and never WTC-OAD subjects, BMI, blood pressure, and HDL were found to be significantly higher at time of REAP-S compared to immediately post-9/11, Table 1. Similarly, their FEV_1%Pred_, HDL, LDL, total cholesterol, and triglycerides were significantly lower at time of REAP-S, and FVC_%Pred_ was not significantly different. WTC-OAD cases had significantly higher BMI, blood pressure, and triglycerides, and lower FEV_1%Pred_, FVC_%Pred_ at 1st post 9/11 and at the time of REAP-S assessment compared to those who never developed WTC-OAD. Subjects with WTC-OAD had an elevated total cholesterol compared to those that never developed WTC-OAD at their 1st post-9/11 assessment. In contrast, at the time of the REAP-S questionnaire, those subjects with WTC-OAD had lower total cholesterol, Table 1.

### REAP-S questionnaire responses

Length of time between initial post 9/11 assessment and REAP-S administration was (mean ± SD) 16.59 ± 0.49 years. The study cohort had a mean ± SD REAP-S score of 29.46 ± 4.22. Subjects with WTC-OAD had significantly lower mean REAP-S score of 28.99 ± 4.37 compared to those who never developed WTC-OAD with 29.60 ± 4.17; p < 0.01. In contrast, 50% of our study cohort often eat more than the recommended amount of meat per day (Q7), 79.30% rarely drink sugary drinks (Q13), 48.80% rarely eat processed meats (Q8), 48.50% rarely eat fried foods (Q9), and 46.40% rarely eat snacks (Q10), Table 2. WTC-OAD cases had significantly higher reported consumption of processed meat (Q8) and sugary drinks (Q13), and decreased intake of grain products (Q3), vegetables (Q5), and fried foods (Q9). WTC-OAD also skipped breakfast more often (Q1), ate out more frequently (Q2), and did not feel well as often to shop or cook (Q15) (p < 0.05), Table 2.

**Table 2:** Nutrition Questions Incorporated into the WTC-HP Annual Questionnaire

Item	All	Ever WTC-OAD	Never WTC-OAD	p
1. How willing are you to make changes in your eating habits in order to be healthier?^a^				
1 Very willing	2096 (52.2)	473 (51.4)	1626 (62.5)	0.129
2	1105 (27.5)	239 (26)	866 (28)	
3	6565 (16.3)	173 (18.8)	483 (15.6)	
4	110 (2.7)	22 (2.4)	88 (2.8)	
5 Not at all willing	49 (1.2)	14 (1.5)	35 (1.1)	
2. Are you willing to answer 15 questions about your diet?^b^				
Yes	4015 (100)	–	–	–
In an average week, how often do you:				
1. Skip breakfast?				
Usually/often (1)	839 (20.9)	215 (23.3)	624 (20.2)	0.030
Sometimes (2)	1180 (29.4)	281 (30.5)	899 (29.1)	
Rarely/never (3)	1996 (49.7)	425 (46.1)	1571 (50.8)	
2. Eat 4 or more meals from sit-down or take out restaurants?				
Usually/often (1)	532 (13.3)	135 (14.5)	398 (12.9)	0.016
Sometimes (2)	1182 (29.4)	297 (32.3)	885 (28.6)	
Rarely/never (3)	2301 (57.3)	490 (53.2)	1811 (58.5)	
3. Eat less than 2 servings of whole grain products or high fiber starches a day? Serving = 1 slice of 100% whole grain bread; 1 cup whole grain cereal like Shredded Wheat, Wheaties, Grape Nuts, high fiber cereals, oatmeal, 3–4 whole grain crackers, ½ cup brown rice or whole wheat pasta, boiled or baked potatoes, yuca, yams or plantain				
Usually/often (1)	744 (18.5)	176 (19.1)	568 (18.4)	0.016
Sometimes (2)	1624 (40.4)	404 (43.9)	1220 (39.4)	
Rarely/never (3)	1647 (41)	341 (37)	1306 (42.2)	
4. Eat less than 2 servings of fruit a day? Serving = ½ cup or 1 med. fruit or ¾ cup 100% fruit juice				
Usually/often (1)	1016 (25.3)	246 (26.7)	770 (24.9)	0.191
Sometimes (2)	1682 (41.9)	395 (42.9)	1287 (41.6)	
Rarely/never (3)	1317 (32.8)	280 (30.4)	1037 (33.5)	
5. Eat less than 2 servings of vegetables a day? Serving = ½ cup vegetables, or 1 cup leafy raw vegetables				
Usually/often (1)	618 (15.4)	169 (18.3)	449 (14.5)	< 0.001
Sometimes (2)	1603 (39.9)	394 (42.8)	1209 (39.1)	
Rarely/never (3)	1794 (44.7)	358 (38.9)	1436 (46.4)	
6. Eat or drink less than 2 servings of milk, yogurt, or cheese a day? Serving = 1 cup milk or yogurt; 1½–2 oz cheese				
Usually/often (1)	826 (20.6)	209 (22.7)	617 (19.9)	0.125
Sometimes (2)	1491 (37.1)	344 (37.4)	1147 (37.1)	
Rarely/never (3)	1698 (42.3)	368 (40)	1330 (43)	
7. Eat more than 8 oz (see sizes below) of meat, chicken, turkey or fish per day? Note: 3 oz of meat or chicken is the size of a deck of cards or ONE of the following: 1 regular hamburger, 1 chicken breast or leg (thigh and drumstick), or 1 pork chop				
Usually/often (1)	2008 (50)	468 (50.8)	1540 (49.8)	0.705
Sometimes (2)	1450 (36.1)	322 (35)	1128 (36.5)	
Rarely/never or rarely eat meat, chicken, turkey or fish (3)	557 (13.9)	131 (14.2)	426 (13.8)	
8. Use regular processed meats (like bologna, salami, corned beef, hotdogs, sausage or bacon) instead of low fat processed meats (like roast beef, turkey, lean ham; low-fat cold cuts/hotdogs)?				
Usually/often (1)	271 (6.7)	80 (8.7)	191 (6.2)	0.001
Sometimes (2)	1784 (44.4)	432 (46.9)	1352 (43.7)	
Rarely/never or rarely (3)	1960 (48.8)	409 (44.4)	1551 (50.1)	
9. Eat fried foods such as fried chicken, fried fish, French fries, fried plantains, tostones or fried yuca?				
Usually/often (1)	174 (4.3)	54 (5.9)	120 (3.9)	0.024
Sometimes (2)	1895 (47.2)	417 (45.3)	1478 (47.8)	
Rarely/never (3)	1946 (48.5)	450 (48.9)	1496 (48.4)	
10. Eat regular potato chips, nacho chips, corn chips, crackers, regular popcorn, nuts instead of pretzels, low-fat chips or lowfat crackers, air-popped popcorn?				
Usually/often (1)	338 (8.4)	79 (8.6)	259 (8.4)	0.111
Sometimes (2)	1815 (45.2)	389 (42.2)	1426 (46.1)	
Rarely/never or Rarely eat these snack foods (3)	1862 (46.4)	453 (49.2)	1409 (45.5)	
11. Add butter, margarine or oil to bread, potatoes, rice or vegetables at the table?				
Usually/often (1)	1059 (26.4)	252 (27.4)	807 (26.1)	0.290
Sometimes (2)	1619 (40.3)	382 (41.5)	1237 (40)	
Rarely/never (3)	1337 (33.3)	287 (31.2)	1050 (33.9)	
12. Eat sweets like cake, cookies, pastries, donuts, muffins, chocolate and candies more than 2 times per day				
Usually/often (1)	678 (16.9)	165 (17.9)	513 (16.6)	0.432
Sometimes (2)	1676 (41.7)	369 (40.1)	1307 (42.2)	
Rarely/never (3)	1661 (41.4)	387 (42)	1274 (41.2)	
13. Drink 16 oz or more of non-diet soda, fruit drink/punch or Kool-Aid a day? Note: 1 can of soda = 12 oz				
Usually/often (1)	259 (6.5)	72 (7.8)	187 (6)	0.001
Sometimes (2)	573 (14.3)	159 (17.3)	414 (13.4)	
Rarely/never (3)	3183 (79.3)	690 (74.9)	2493 (80.6)	
14. You or a member of your family usually shops and cooks rather than eating sit-down or take-out restaurant food?				
Yes	3673 (91.5)	831 (90.2)	2842 (91.9)	0.120
15. Usually feel well enough to shop or cook				
Yes	3876 (96.5)	872 (94.7)	3004 (97.1)	< 0.001

Values represented by N (%); x^2^ was done for comparison between Ever WTC-OAD and Never WTC-OAD. Significant values reported if p < 0.05

^a^This is the 16th REAP-S question

^b^If participant answers “Yes”, the participant will be prompted to answer the 15 questions from REAP-S

### Quality of diet assessed by REAP-S

**Fig. 2:**
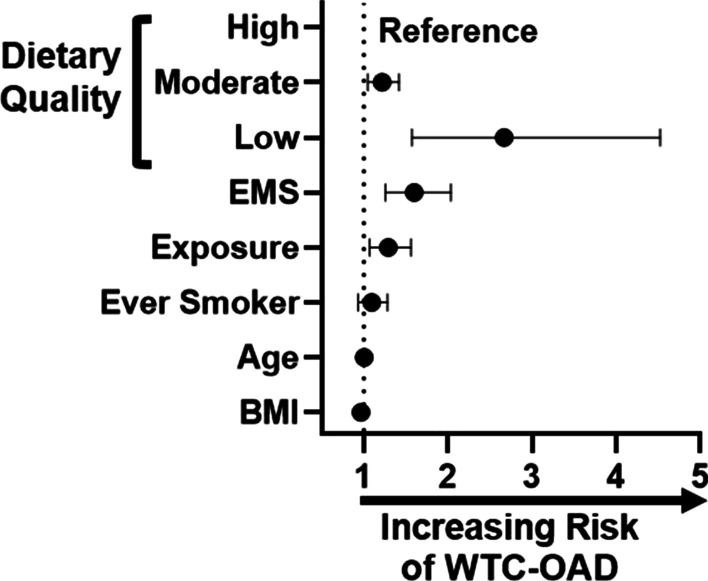
REAP-S Score Modeling of Associated WTC-OAD. Low nutrition is a significant risk factor of developing WTC-OAD. The model was adjusted for age on 9/11, ever smoking, BMI post 9/11, exposure intensity, and job description

Low-dietary quality was significantly associated with 2.67 odds (95% CI [1.57, 4.52]; p < 0.01) of developing WTC-OAD whereas moderate-dietary quality was associated with 1.22 odds (95% CI [1.05, 1.42]; p = 0.01), when comparing to high-dietary quality as a reference group, Fig. 2. Increasing BMI had a small but significant protective odds ratio of 0.97 (95% CI [0.95, 0.98]; p < 0.01). Job description was significant, at 1.60 odds (95% CI [1.26, 2.03]; p < 0.01). Exposure intensity was a time-dependent risk factor, with 1.29 odds (95% CI [1.07, 1.56]; p = 0.01). Age at 9/11 and smoking were not significant risk factors in this model. Overall, job description, exposure, and BMI were found to have significant odds of developing WTC-OAD, while age at 9/11 and smoking were not, Fig. 2.

*Dietary quality subgroups and lung function* of those with low-, moderate-, or high-dietary quality are shown in Table 3. Mean FEV_1%Pred_ and FVC_1%Pred_ at both time points are significantly higher in those with higher dietary quality compared to those with lower dietary quality (p < 0.05). FEV_1_/FVC ratio was not significantly associated with dietary quality at either timepoint, Table 3.

**Table 3:** Dietary quality subgroup analysis

Time	Spirometry	Dietary quality			p
		Low (N = 61)	Moderate (N = 1894)	High (N = 2060)	
1st Post-9/11	FEV_1%Pred_	93.57 (15.34)	96.28 (14.01)	97.61 (13.94)	< 0.01
	FVC_%Pred_	90.30 (13.28)	91.95 (12.15)	92.81 (12.09)	0.04
	FEV_1_/FVC	0.83 (0.05)	0.84 (0.06)	0.84 (0.06)	0.79
REAP-S	FEV_1%Pred_	86.45 (19.87)	91.95 (14.28)	94.12 (14.08)	< 0.001
	FVC_%Pred_	85.82 (14.86)	90.58 (12.32)	92.64 (12.51)	< 0.001
	FEV_1_/FVC	0.77 (0.08)	0.77 (0.06)	0.77 (0.05)	0.46

All values displayed as mean(SD). p-value calculated by ANOVA

### Processed meat, sugary drinks, and vegetable intake impacted the odds of developing WTC-OAD

**Fig. 3:**
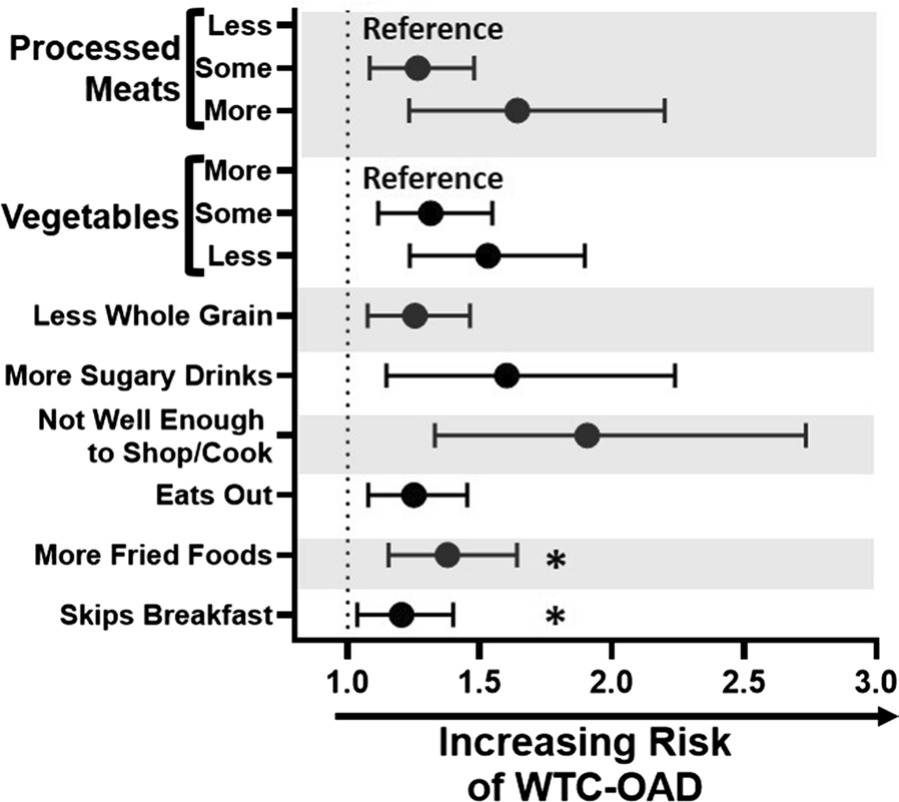
Food Groups Modeling of Associated WTC-OAD. Food groups represented by its corresponding REAP-S questions identified consumption of less vegetables, more processed meats, and some sugary drinks as significant risk factors. The model was adjusted for age on 9/11, ever smoking, BMI post 9/11, exposure intensity, and job description. *Not significant under Bonferroni correction of p < 0.005, but significant for p < 0.05

Assessment of individual REAP-S questions highlighted that WTC-OAD was more likely in subjects with increased consumption of processed meats (Q8) and sugary drinks (Q13), and decreased intake of vegetables (Q5), Table 2 and Fig. 3. Additionally, there was a dose response seen with increasing intake of processed meats (OR 1.64 (95% CI [1.23, 2.19]; p = 0.001) and 1.27 (95% CI [1.08, 1.48]; p = 0.003)) and less vegetables (OR 1.53 (95% CI [1.24, 1.90]; p < 0.001) and 1.31 (95% CI [1.12, 1.55]; p = 0.001)). Less whole grain consumption is also associated with higher risk of WTC-OAD (Q3), 1.26 (95% CI [1.08, 1.46]; p = 0.004). WTC-OAD subjects trended towards increased fried food intake but these measures were not significant after Bonferroni correction (p = 0.006), Table 2 and Fig. 3.

### Dietary habit assessment showed that not being well enough to cook*,* skipping breakfast, and eating out increase odds of WTC-OAD

Not feeling well enough to cook (Q15) increased odds of developing WTC-OAD by 1.91 (95% CI [1.33, 2.73]; p < 0.001) whereas skipping breakfast (Q1) was 1.20 (95% CI [1.04, 1.40]; p = 0.015). Eating out (Q2) also had odds of 1.25 (95% CI [1.08, 1.45]; p = 0.003), Table 2.

### AGE rich foods confer a higher likelihood of developing WTC-OAD

**Fig. 4:**
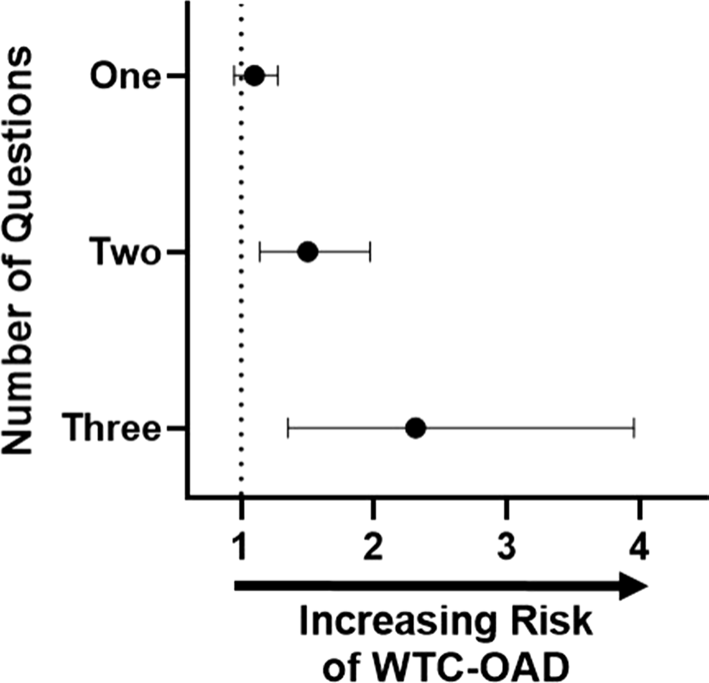
AGEs and their Association with WTC-OAD. Forest plot of AGE in dietary habit represented by frequency of “usually/often” answers to questions on foods high in AGE (fried foods, processed meats, meats). Having a higher dietary habit of AGE is significantly associated with risk of WTC-OAD

Using data adapted from Uribarri et al., we summarized the amount of AGE in food groups represented in REAP-S, Additional file 2: Figure S1 [78]. Fried foods (3971.86 kU/serving), processed meats (3925.89 kU/serving), and meats (3687.58 kU/serving) were identified as having the highest AGEs per serving. Sugary foods and drinks (7.2 kU/serving) do not naturally have high level of AGEs but instead cause high levels of endogenous AGEs. Frequency of eating foods highest in AGEs, meat (Q7), processed meats (Q8), and fried foods (Q9), was assessed by logistic regression model adjusted for age, smoking, BMI, exposure, and job description. An AGE-rich exposure response gradient was identified with the odds of developing WTC-OAD: not significantly increased in participants answering usual/often consumption of one AGE-rich food group, significantly increased in participants answering usual/often consumption to any two AGE-rich food groups, 1.50 (95% CI [1.14, 1.97]; p = 0.04), and highly significant in those answering usual/often consumption to all three AGE-rich food groups, 2.31 (95% CI [1.35, 3.95]; p = 0.002), Fig. 4.

## Discussion

This observational, prospective study of dietary phenotyping was successfully implemented at the FDNY WTC-HP annual monitoring exam. Dietary quality was correlated to FEV_1_ and FVC even immediately after 9/11, and persisted at REAP-S. Since we assume that diet is constant throughout adult life, this could be due to the combined effect of dietary quality and WTC exposure. This is supported by our findings in which more frequent consumption of sugary drinks, processed meats, and decreased intake of vegetables and whole grains were identified as key components in development of WTC-OAD. Subjects with AGE-rich diets were also significantly more likely to develop WTC-OAD.

Our findings parallel prior studies that have displayed the harmful role of processed meats in the increased risk of developing COPD due to its high level of pro-inflammatory AGE levels [89–92]. AGE formation via glycoxidation is promoted by the high temperatures and low moisture environments utilized in cooking meat, processed meat products, and fried foods [78, 93–95]. Associations with certain food groups are important because high levels of AGEs are linked to pathogenic effects, including the ability to promote high levels of oxidative stress and inflammation [78, 94].

Although sugary drinks are relatively low in AGEs, they are a prominent source of high fructose corn syrup [27]. The fructose can indirectly increase AGE intake because of its formation and accumulation of endogenous AGEs [27]. This could be a reason as to why sugary drinks have been associated with bronchitis and asthma in children, and increase likelihood of WTC-OAD [96, 97]. In contrast, carbohydrates and whole grains contain less AGE [78, 95]. Similar to our results, other studies have found that increased whole grain intake, part of a prudent dietary pattern, were associated with a reduced risk of developing COPD [13]. Moreover, it was positively associated with FEV_1_ and negatively associated with COPD symptoms [98].

Although we showed that low intake of vegetables increased odds of developing WTC-OAD, there was no significant association found with fruit intake. While our results resonated with some studies on low intake of fruits and vegetables, others advocated for increased fruit intake, or found no difference in COPD [78, 99–104]. Although fruits and vegetables are relatively low in AGEs, they could confer antioxidant benefit and lower inflammation in diseases such as COPD. A randomized control trial focused on increased antioxidant intake through fruits and vegetables found that it could even help regain FEV_1_ in COPD patients [102].

In contrast to our prior work, increasing BMI had a 3.6% decreased likelihood of developing WTC-OAD. One reason for this difference could be that while our prior work focused on firefighters, we now also investigate EMS 1st responders. Prior studies have shown that the firefighter and EMS cohort express different patterns of lung function decline, even after adjusting for BMI [18]. This expanded cohort potentially reflects concordance with studies showing obesity’s protective effect against mortality in COPD patients [105, 106]. This could also be a result of a healthy worker effect and the imperfect utilization of BMI to define obesity in firefighters with rigorous physical job requirements [107]. In addition, we optimized our model by adjusting for confounding of WTC-OAD cases by using BMI at the time of diagnosis, whereas for subjects that never developed WTC-OAD, BMI at REAP-S was used. Nevertheless, the results of the final model did not change significantly even when we also assessed the effects of BMI at the same time point (1st post 9/11 and at REAP-S respectively). Moroever, we found that triglycerides decreased from post 9/11 to REAP-S. This could be an effect of the close monitoring that these patients received and/or other confounder such as triglyceride-lowering medications such as statin therapy in as per 2018 American Heart Association/American College of Cardiology guidelines [108]. Future studies could help differentiate the paradoxical effect of obesity vs. the healthy worker phenomena.

There are several limitations to our investigation. Dietary habits and exposure are subject to self-reporting bias. Bias assessment has been performed on the FDNY WTC cohort, and found that self-reported asthma and exposure were consistent across several studies, and that significant findings were minimally affected by potential bias [109]. Thus, extension of the reliability of this data to our study is reasonable. We also assume that dietary habits remain relatively constant throughout a person’s adult life, an assumption supported by several large studies [86, 88, 110]. Additionally, REAP-S is a brief dietary instrument that limits our ability to differentiate subtypes of food consumed. Protein intake does not differentiate between beans, poultry, or red meat. Firefighters had pre-employment physical health assessments to ensure that they did not have pre-existing OAD, the pulmonary function for EMS prior to 9/11 was also assessed but these 1st responders are not subject to the same standards. Therefore, our results are limited to correlation of dietary quality and OAD.

Another possible limitation is that WTC-OAD subjects were more likely to be retired compared to those who never developed WTC-OAD. We also identified that not being well enough to shop and cook and frequent eating out had strong associations with WTC-OAD. Since this was assessed at a later time point, it is unclear if this reflects the burden of concurrent WTC-OAD. One study has found that physical factors with COPD patients such as being too tired to cook resulted in the shift in eating meals that have been easily prepared [111]. However, this is an important finding that highlights a potential barrier to access to healthier diets in this population. The limiting lifestyle imposed by WTC-OAD could further perpetuate an unforgiving cycle of lower dietary quality and worsening disease.

This study identifies risk factors of worsening OAD, and demonstrates the potential for intervention. Further research is needed to determine if implementation of a diet focused on decreasing AGE-rich foods, increasing antioxidant intake, and targeting weight loss could prevent the development of WTC-OAD or in those with WTC-OAD, reverse or slow its progression. Our ongoing randomized clinical trial, the Food Intake Restriction for Health Outcome Support and Education (FIREHOUSE) Trial, aims to assess the effects of technology-assisted social cognitive behavioral therapy and a low-caloric Mediterranean diet on the progression of WTC-LI.

In summary, this observational study successfully used REAP-S to identify both low-dietary quality and AGE abundant foods as predictive of developing lung disease in this WTC-exposed population. Our findings indicate the potential impact of future research using dietary interventions not just in the FDNY WTC-HP, but also in other OAD cohorts, with or without WTC-exposure.

## Supplementary Information

**Additional file 1: Table S1.** Full Nutrition Questions. All of the nutrition questions that were incorporated into the WTC-HP annual questionnaire.

**Additional file 2: Figure S1.** Assessment of AGEs in REAP-S Food Groups. REAP-S identified food groups (fried foods, processed meats, and meats) that have the highest amounts of AGE (kU/serving) adapted from Uribarri et al. [78]

## Data Availability

Sharing of human data is governed by the World Trade Center (WTC) Clinical Center of Excellence program maintained by the Fire Department of New York (FDNY). All investigators will need to enter into a data use agreement with the FDNY WTC Clinical Center of Excellence. Additional information about this database may be obtained through Dr. David Prezant. He can be reached by email at prezand@fdny.nyc.gov.

## Abbreviations

AGE: Advanced glycation end-products
AHR: Airway hyperreactivity
BMI: Body Mass Index
CI: Confidence interval
DBP: Diastolic blood pressure
EMS: Emergency Medical Services
FDNY: Fire Department of the City of New York
FEV_1_: Forced expiratory volume over 1 s
FFQ: Food Frequency Questionnaire
FVC: Forced vital capacity
HDL: High density lipoprotein
HR: Hazards ratio
LDL: Low density lipoprotein
LLN: Lower limit of normal
MetSyn: Metabolic syndrome
NHANES: National Health and Nutrition Examination Survey
OR: Odds ratio
PFT: Pulmonary function test
PM: Particulate matter
PUFA: Polyunsaturated fatty acids
REAP-S: Rapid Eating and Activity Assessment for Patients-Short Version
SBP: Systolic blood pressure
SD: Standard deviation
US: United States
WTC: World Trade Center
WTC-HP: WTC-Health Program
WTC-LI: WTC-Lung Injury
WTC-OAD: WTC-obstructive airways disease
WTC-PM: WTC-particulate matter
