# Supplementary material for: Dietary phenotype and advanced glycation end-products predict WTC-obstructive airways disease: a longitudinal observational study
Source: Respir Res. 2021 Jan 18;22:19. doi: 10.1186/s12931-020-01596-6 (PMC7812653; doi:10.1186/s12931-020-01596-6)
Supplement: Supplementary file 1 — Additional file 1: Table S1. Full Nutrition Questions. All of the nutrition questions that were incorporated into the WTC-HP annual questionnaire. [file 12931_2020_1596_MOESM1_ESM.pptx]

## Slide 1
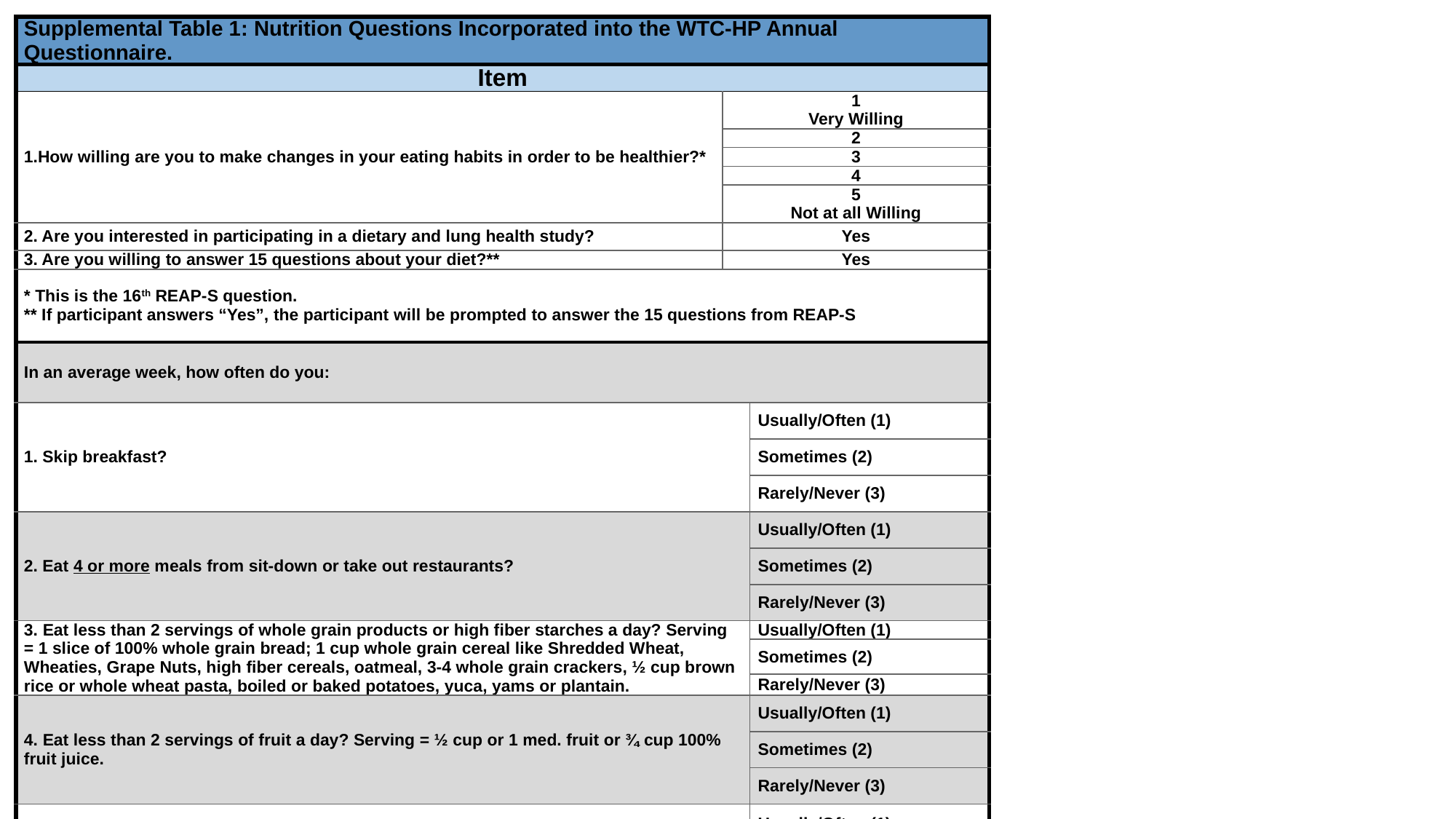

| Supplemental Table 1: Nutrition Questions Incorporated into the WTC-HP Annual Questionnaire. | | |
| --- | --- | --- |
| Item | | |
| 1.How willing are you to make changes in your eating habits in order to be healthier?\* | 1 Very Willing | |
| | 2 | |
| | 3 | |
| | 4 | |
| | 5 Not at all Willing | |
| 2. Are you interested in participating in a dietary and lung health study? | Yes | |
| 3. Are you willing to answer 15 questions about your diet?\*\* | Yes | |
| \* This is the 16th REAP-S question. \*\* If participant answers “Yes”, the participant will be prompted to answer the 15 questions from REAP-S | | |
| In an average week, how often do you: | | |
| 1. Skip breakfast? | | Usually/Often (1) |
| | | Sometimes (2) |
| | | Rarely/Never (3) |
| 2. Eat 4 or more meals from sit-down or take out restaurants? | | Usually/Often (1) |
| | | Sometimes (2) |
| | | Rarely/Never (3) |
| 3. Eat less than 2 servings of whole grain products or high fiber starches a day? Serving = 1 slice of 100% whole grain bread; 1 cup whole grain cereal like Shredded Wheat, Wheaties, Grape Nuts, high fiber cereals, oatmeal, 3-4 whole grain crackers, ½ cup brown rice or whole wheat pasta, boiled or baked potatoes, yuca, yams or plantain. | | Usually/Often (1) |
| | | Sometimes (2) |
| | | Rarely/Never (3) |
| 4. Eat less than 2 servings of fruit a day? Serving = ½ cup or 1 med. fruit or ¾ cup 100% fruit juice. | | Usually/Often (1) |
| | | Sometimes (2) |
| | | Rarely/Never (3) |
| 5. Eat less than 2 servings of vegetables a day? Serving = ½ cup vegetables, or 1 cup leafy raw vegetables. | | Usually/Often (1) |
| | | Sometimes (2) |
| | | Rarely/Never (3) |
| 6. Eat or drink less than 2 servings of milk, yogurt, or cheese a day? Serving = 1 cup milk or yogurt; 1½ - 2 ounces cheese. | | Usually/Often (1) |
| | | Sometimes (2) |
| | | Rarely/Never (3) |
| 7. Eat more than 8 ounces (see sizes below) of meat, chicken, turkey or fish per day? Note: 3 ounces of meat or chicken is the size of a deck of cards or ONE of the following: 1 regular hamburger, 1 chicken breast or leg (thigh and drumstick), or 1 pork chop. | | Usually/Often (1) |
| | | Sometimes (2) |
| | | Rarely/Never or Rarely eat meat, chicken, turkey or fish (3) |
| 8. Use regular processed meats (like bologna, salami, corned beef, hotdogs, sausage or bacon) instead of low fat processed meats (like roast beef, turkey, lean ham; low-fat cold cuts/hotdogs)? | | Usually/Often (1) |
| | | Sometimes (2) |
| | | Rarely/Never or Rarely eat processed meats (3) |
| 9. Eat fried foods such as fried chicken, fried fish, French fries, fried plantains, tostones or fried yuca? | | Usually/Often (1) |
| | | Sometimes (2) |
| | | Rarely/Never (3) |
| 10. Eat regular potato chips, nacho chips, corn chips, crackers, regular popcorn, nuts instead of pretzels, low-fat chips or lowfat crackers, air-popped popcorn? | | Usually/Often (1) |
| | | Sometimes (2) |
| | | Rarely/Never or Rarely eat these snack foods (3) |
| 11. Add butter, margarine or oil to bread, potatoes, rice or vegetables at the table? | | Usually/Often (1) |
| | | Sometimes (2) |
| | | Rarely/Never (3) |
| 12. Eat sweets like cake, cookies, pastries, donuts, muffins, chocolate and candies more than 2 times per day. | | Usually/Often (1) |
| | | Sometimes (2) |
| | | Rarely/Never (3) |
| 13. Drink 16 ounces or more of non-diet soda, fruit drink/punch or Kool-Aid a day? Note: 1 can of soda = 12 ounces | | Usually/Often (1) |
| | | Sometimes (2) |
| | | Rarely/Never (3) |
| 14. You or a member of your family usually shops and cooks rather than eating sit-down or take-out restaurant food? | | Yes |
| 15. Usually feel well enough to shop or cook. | | Yes |
