# Supplementary material for: Dietary phenotype and advanced glycation end-products predict WTC-obstructive airways disease: a longitudinal observational study
Source: Respir Res. 2021 Jan 18;22:19. doi: 10.1186/s12931-020-01596-6 (PMC7812653; doi:10.1186/s12931-020-01596-6)
Supplement: Supplementary file 2 — Additional file 2: Figure S1. Assessment of AGEs in REAP-S Food Groups. REAP-S identified food groups (fried foods, processed meats, and meats) that have the highest amounts of AGE (kU/serving) adapted from Uribarri et al. [78] [file 12931_2020_1596_MOESM2_ESM.pptx]

## Slide 1
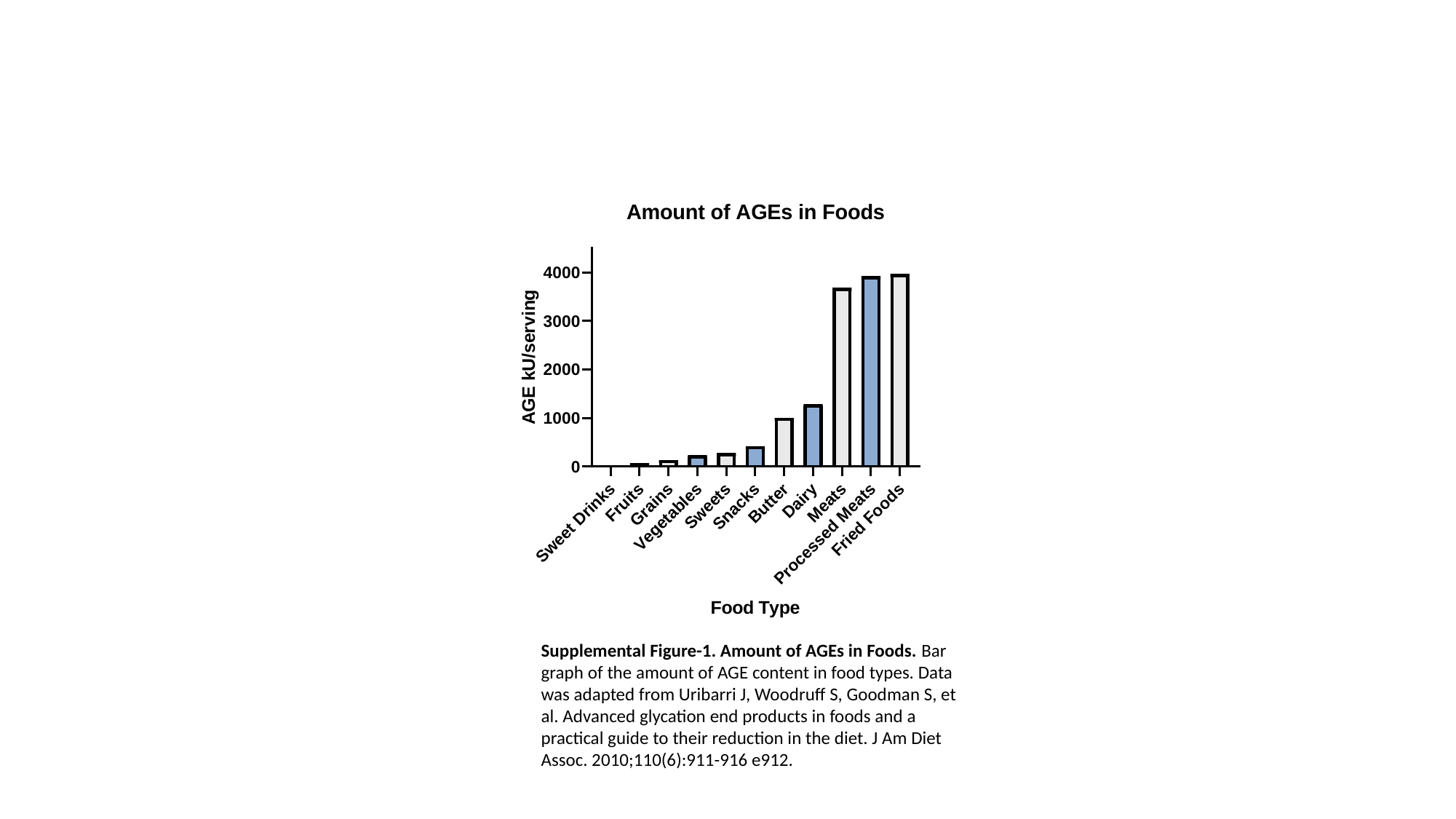

Supplemental Figure-1. Amount of AGEs in Foods. Bar graph of the amount of AGE content in food types. Data was adapted from Uribarri J, Woodruff S, Goodman S, et al. Advanced glycation end products in foods and a practical guide to their reduction in the diet. J Am Diet Assoc. 2010;110(6):911-916 e912.
